# Fault analysis on deep groove ball bearing using ResNet50 and AlexNet50 algorithms

**DOI:** 10.1038/s41598-025-97410-8

**Published:** 2025-04-15

**Authors:** Vedant Jaiswal, Narendiranath Babu T, Pandiyan Murugan, Rama Prabha D

**Affiliations:** 1 School of Mechanical Engineering, Vellore Institute of Technology (VIT), Vellore, 632 014 India; 2 School of Electrical Engineering, Vellore Institute of Technology (VIT), Vellore, 632 014 India

**Keywords:** Ball bearing, Artificial neural network, Fault classification, Machine learning, Deep neural network, Support vector machine, Mechanical engineering, Aerospace engineering

## Abstract

Deep Groove Ball Bearings (DGBBs) serve multipurpose and are used for the propeller shaft movement and applications based on revolving. They have great applications in industry related to axial and radial loads. The major risk factors are faults in bearings. Data analyzed for faults in the DGBBs help us conclude that there are 4 types of bearing faults. For instance, Excluding HB- Healthy Bearing, there are CF- Case Fault, BF- Ball Fault, IRF- Inner Ring Fault, and ORF- Outer Ring Fault. The input parameters are represented by using 14 features in the evaluation. Next, a feature ranking method is established to classify the bearing fault and contribution of each of the features is used as input conditions. It displays the involvement value for each of the 14 parameters. Automatic fault classification has been done by Artificial Neural Networks (ANN). Training on various algorithms is performed, noting and storing the probability of correct prediction for comparison. The probability of correct predictions decreases as the number of samples representing faults increases. A high efficiency of around 97.9% has been achieved for the Resnet50 algorithm. The classifier learner achieved an accuracy of 97% using the neural network, followed by the decision tree and discriminant analysis.

## Introduction

ANN was used for the analysis of the cryogenic ball bearing resulting in an accuracy of 90%+. The tensor flow python library was used for checking the testing values. RUL prediction model was used in the feature extraction method^1^.

Due to the advancement in the infotainment technology, data-driven approaches like machine learning are being increasingly used for the status diagnosis. The Accuracy is paramount as it indicates the quality of the data and sensor selection. Unsupervised machine learning has a label issue but it helps for improvement in evaluating bearing conditions^2^.

Unsupervised learning is called a non—labelled data set. Its results were evaluated on complex and high dimensional problems. The use of clustering Algorithm for fault diagnosis for the ball bearing results in analysis of complex input with noise and reductance and improved analysis within all the 3 axes of the reading^3^.

The prostate cancer was analysed with an algorithm called alexnet50 form CNN deep neural network. It compared the 3D training value with the real time provided data to accurately detect the cancers and save life. The provided accuracy rate was about 92% to 93% including parameters such as RMS and mean absolute distances for finding the accuracy^4^.

Polymer ball bearings were used for vibration analysis. Model was developed with understanding the complex shapes and size of the bearing and helped with providing accurate layer to layer production of the component^5^.

The importance of health monitoring of machines in the industry is helping in preventing the loss of time, space, and money. So, it improves strategy that affects the period of maintenance. Predictive maintenance was used for conditional monitoring techniques to check and provide warning for possible faults time ahead of failure. Its major role reduces the time after the repair. It helps in understanding real-time machine status and extracting data with time domain in 3 directions^6^.

Fatigue Life theory method was chosen for the calculation of material and forces. The life of bearing was considered with this model which was trained for 60 + applications for ball bearing, resulting in providing high order of accuracy^7^.

The bearing is important for revolving machine components due to the very high failure rate. If fault is not found, then it can affect the whole performance. It tells about methods for extracting the ball bearing fault. It detects faults within the range of wavelet packet transforms_(WPT). The ML models are used for condition and enhanced defect diagnostics and their feature extraction^8^.

It shows that machine learning and artificial intelligence are very computational intelligence methods. AI is used for prediction and analysis of the damages with the use of pre-treated experimental data used for wear of the contacting surfaces as a criterion of damages used for a lifetime prediction. Neural Network structures are to be implemented for learning from the given data and describing wear formulas and ANN fuzzy inference systems to impose a hand on meaningful rules. Performance, generalising ability, complexity, and time consumption are fundamental elements for evaluating artificial intelligence methods^9^.

Machine components fail for various reasons. Their habits and paths are created from their data set from their different applications. There are paths created for a component to prevent the noise and disturbance. Then, clustering was used and RUL prediction models were also required to get the General Log-Linear Weibull (GLL-Weibull) distribution^10^.

Angular contact ball bearings (ACBBs) were used for mechanical systems related to revolving machining that determined the inner structure of the ACBB. Its motion related activities were captured by the process called hysteresis, friction, heat dissipation, etc. Electro hydromics will be utilised for providing lubrication with the condition that the models will be trained for accuracy detection^11^.

Fully Conventional Neural Networks (FCNNs) have caused segmentation models to emerge which require a large amount of time that results in complex architectures. It tells about the efficiency of the model through the accuracy segment of the 3D visual processing. To solve this, 2D and 3D self-adaptive AdaEn-Net algorithm is proposed for image processing which incorporates volume data. 2D extracts information, whereas 3D exploits intra-slice information. This fully exploits the volumetric information and searches for a high-grade and efficient model. AdaEn-Net ranks 9 out of 297 submissions and ranks 2 within the top 8 submissions^12^.

Deep neural network algorithms such as Resnet50 and Xception were used, where maximum accuracy was achieved by combining all the skin related defects such as melanoma, etc. An astonishing 10,000+ sample data were collected from multiple patients for achieving an accuracy of 97.8%. For feature extraction analysis of variance (ANOVA) was used^13^.

The new technology in the manufacturing industry revolving from 3.0 to 4.0 is its predictive maintenance. The fault components can be identified easily and fatigue failure can be prevented. Multiple algorithms are required to preventing equipment failure. Al algorithms such as Autoencoders, Artificial Neural Network (ANN), and Random Forest (RF) classification helped for accuracy detection with various types of faulty bearing data that is supported by CWRU^14^.

Experts examined ball bearing fault classification through vibration signals, introducing a hybrid model that integrated Fuzzy Min–Max (FMM) with Random Forest (RF). The study highlighted the effectiveness of the FMM-RF model, achieving 99.9% accuracy on benchmark datasets and 99.8% on real-world datasets^15^.

Predictive maintenance techniques were performed for an essential role for avoiding equipment failure and ensuring the efficiency of production facilities. They cover the theory and application of various basic techniques, vibration analysis, ultrasonic analysis for small fault defects and thermal anomalies^16^.

A model of ball bearing was proposed which was kept in motion for a prolonged period of time. The model considered, after a period of run, the result for deformation in the bearing structure including wear and tear with the bearing onto the inner ball that revolved providing minimum point of contact between the surfaces. At conditions like this improved the training of the AI algorithm for better and accurate analysis^17^.

The paper utilised a very new model transformation called Resnet—vision, which was then really used for rolling bearing fault analysis. It measured 1D vibration data from the accelerometer sensor for reading. The complex noise and redundancy were reduced from a process SVD that increased the accuracy percentage of the data set^18^.

The provided paper diagnosed the faulty ball bearing with the Deep neural network algorithm. While remaining diagnosed with a common artificial neural network, the provided primary analysis concluded with the following: Support vector machine, etc. The paper’s proven point was from fault feature extraction and based on classification. Survey was conducted through algorithms using CWRU bearing dataset^19^.

The hybrid deep learning model achieved better accuracy in detecting various fault conditions in milling machines, significantly outperforming traditional approaches. The model was validated using acoustic emission data collected from a controlled laboratory milling machine testbed, demonstrating its effectiveness in real-world applications. The method offered a highly accurate and efficient solution for fault detection, enabling reliable predictive maintenance and improved operational efficiency in industrial settings^20^.

Accuracy of the vibration data was noted for a ball bearing with the help of multiple algorithm such as Deep neural network (DNN) and Artificial neural Network (ANN) with the use of MATLAB classifier and convolutional neural network (CNN) model ResNet50 and AlexNet50 for accuracy detection for the predicted and true data. Feature extraction had a total of 14 features in Hand to work on. The confusion matrix and trained network function in MATLAB defined the highest accuracy given by the models for the fault diagnosis of the ball bearing.

### Research gap

Random Forest algorithm was used for predictive analysis of deep groove ball bearings. However, the computational time required for this analysis was not reported in the previous studies. Random Forest is an ensemble learning method that builds multiple decision trees and merges them to get a more accurate and stable prediction. The computational complexity can vary depending on the dataset size, number of trees, and depth of trees.

Convolutional Neural Network (CNN) is an image processing algorithm known for its high accuracy. It uses convolutional layers to automatically and adaptively learn spatial hierarchies of features from images. Despite its effectiveness, the work done on ball bearings using CNN lacked proper accuracy. CNNs are particularly powerful for image classification tasks due to their ability to capture local patterns like edges and textures. CNNs are underutilised for 1D analysis and mainly result in 3D analysis (like Image Processing, etc.,). Despite providing high-accuracy classification with feature extraction from 1D vibration signal input.

Also, the previous analyses did not provide detailed information on the computational time required for their respective processes. This information is crucial for evaluating the efficiency and practicality of these algorithms in real-world applications.

### Main objective

*Data Acquisition*: Beginning with efficient data acquisition method, it is used to collect the vibration data for predictive analysis and fault detection in deep groove ball bearings.

*Implement Feature Selection*: Investigate the effectiveness of the Minimum Redundancy Maximum Relevance (MRMR) feature selection method on improving the classification results of deep learning algorithms in deep groove ball bearing fault analysis.

*Enhance Accuracy*: Improve the accuracy of Convolutional Neural Network (CNN) algorithms in the analysis of deep groove ball bearings, particularly focusing on 1D vibration signal input.

*Evaluate Computational Time*: Assess the computational time required for predictive analysis of deep groove ball bearings using the DL algorithms.

*Compare CNN Algorithms*: Compare the performance of ResNet-50 and AlexNet-50 in terms of accuracy with the traditional methods for vibration analysis of deep groove ball bearings.

## Experimental set-up

**Fig. 1:**
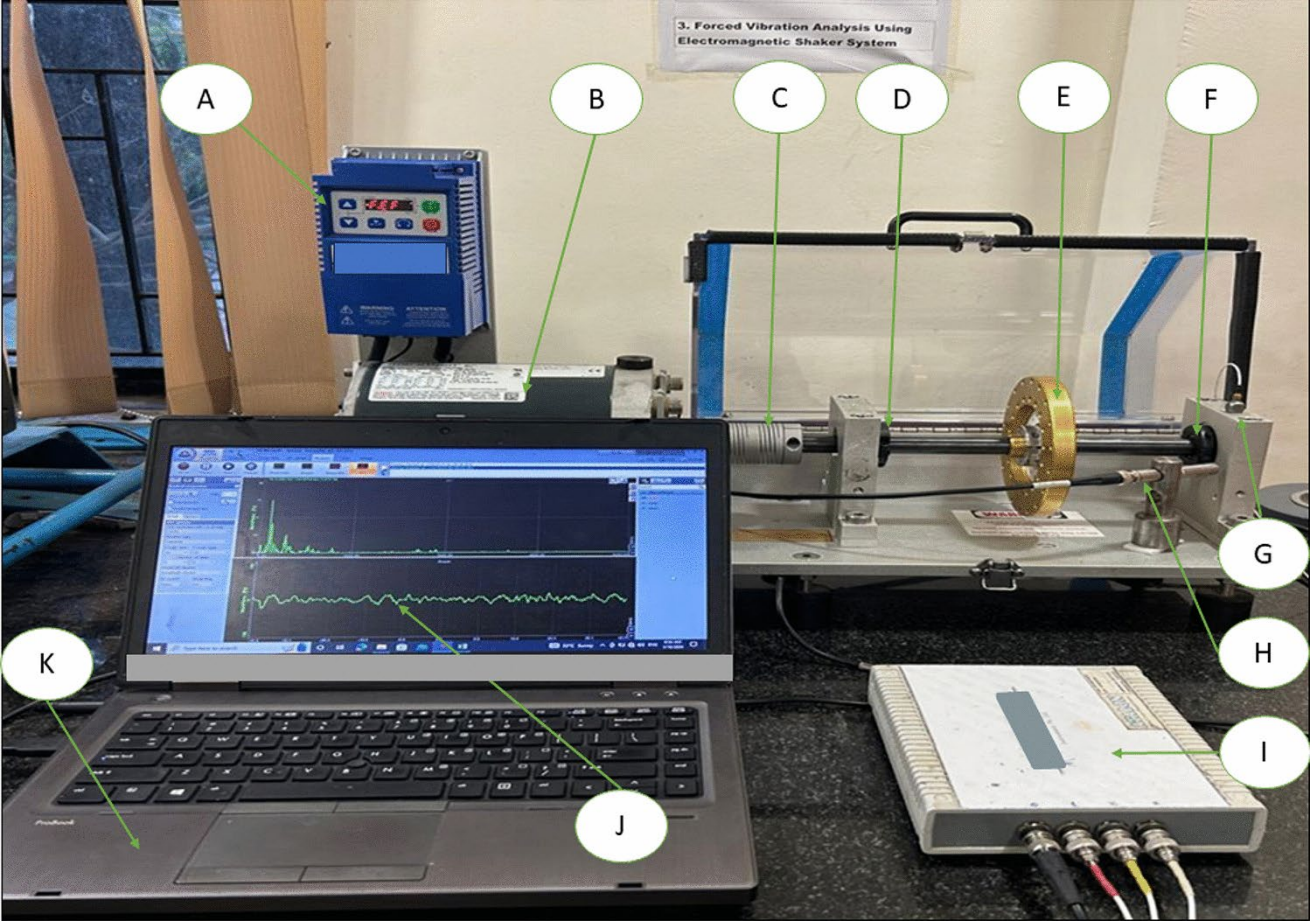
Experimental Set-up. (A) Frequency control Unit: Applying 50 Hz of frequency as a standard given by the Indian electricity. (B) Dc motor: The motor is rotating the ball bearing at a standard of 4000 rpm at a power input of 1/3 HP. (C) Coupling (Flexible coupling): It minimises the occurrence of misalignment effect. It was utilised to absorb shock loads and damped vibration that’s produced between the connecting shafts. It reduced the unwanted disturbance produced by the experiment and increased the Accuracy of the experimental input values. (D) Support bearing (MBER10K) deep groove ball bearing: It was used for the purpose of input values. providing about 5 classes of different bearing. Each of the bearings has unique defects. (E) Balancing Disk: It improved imbalance that was induced by the vibration by controlling the vibration produced and reduced the noise. It increased the signal clarity for the fault bearing. (F) Test bearing: Provided the fault bearing by the company, a total of 5 classes for bearing were given or used for the respective analysis. (G) Triaxial Accelerometer: Its role was to measure vibration for all the three given Gaussian coordinate (X, Y, Z) Direction. The bearing was rotated at a high speed that resulted in vibration, that was transferred to the DAQ system after rectifying noise and transferring analog to digital signal. (H) Microphone: It captures airborne Sound that was produced due to faulty bearing. It then converts the signal into an electrical signal output. It captures the surrounding sound as well hence the experiment was conducted at a silent place without any disturbance. (I) DAQ System (Data Acquisition system): It helps in storing real-time vibration data from the sensor. It supports converting the analog signal vibration data into digital signal vibration data. The reading from the sensors was stored and graphs were plotted through transferring of the data to the Dewesoft software. (J) Then Apply (FFT)- Fourier Transform and Time-Domain Analysis which supports in monitoring the vibration signal and prevents from exceeding the pre-required frequency domain. The Domain was fixed due to Indian standard that is 50 HZ and it was maintained throughout the period of operation. (K) Storage device such as DeweSoft 7.1: It stores the large Set of data within the format while consuming less space. It utilises for measuring the vibration real-time and produces the vibration value into an excel format.

In our roller bearing experiment, we utilized a bearing fault simulator (BFS), a device owned by our institution, specifically designed for bearing fault-related issues is shown in Fig. 1. The BFS provides a stable platform for collecting vibrational data and is encased in a frame that blocks external noise. First the Frequency Control Unit was used to maintain a standard frequency of 50 Hz, in line with Indian electricity standards. The DC motor was operated at 4000 rpm with a power input of 1/3 HP to rotate the ball bearings. Next, a flexible coupling was employed to minimize misalignment effects, absorb shock loads, and dampen vibrations between the connecting shafts, thereby reducing unwanted disturbances and enhancing the accuracy of the experimental input values. Then, support bearings (MBER10K deep groove ball bearings) were utilized, offering five different classes of bearings with distinct defects. Furthermore, a balancing disk was used to control vibration-induced imbalances, reduce noise, and improve signal clarity for fault-bearing analysis. Faulty bearings provided by the company were categorized into five classes for analysis.

This controlled experimental set-up closely simulates real-world industrial conditions. We have tested the models at various rotational speeds (1000 RPM, 1500 RPM, 2000 RPM, and 2500 RPM) to demonstrate their adaptability to changing operating conditions. Noise resilience will achieve through data preprocessing techniques, ensuring strong performance even with synthetic noise. The models were evaluated on various fault types, including inner race, outer race, ball, and combined faults, equipping them to handle diverse industrial fault scenarios. Their flexibility allows for the integration of additional fault types as needed. Our methodology, utilizing vibration signals and advanced deep learning models like ResNet50 and AlexNet50, is applicable to other bearings and machinery. The models remain robust across different environments and bearing types with properly captured vibration data. We recognize the variability in real-world conditions and the necessity for continuous improvement. Future efforts will focus on validating the model in various industrial settings, incorporating complex noise conditions, variable load applications, and expanding the range of fault types.

### Fault bearing

A total of 4 types of faults bearing classes for the process were taken, that were created using EDM.

CF- It can happen due to wear, fatigue or Deformation of the respective bearing case. (High Speed can cause centrifugal force that stresses the case).

BF- It happens due to damages. Such as: Spalling, cracking or pitting. Due to Dirt, less lubrication, micro-cracks.

IRF- It occurs due to the damage to the inner ring of the ball bearing and due to Load Stress caused by the cracks.

ORF- It occurs due to the Damaging of the Outer ring off the deep groove ball bearing and due to Heavy Static Load, Shock Load, Misalignment, Contaminations.

### Technical specifications

Table1 shows experiment parameter details used in the study.

**Table 1:** Details of the experiment parameters.

Parameters	Details
Experimental model	Spectra Quest
Bearing type	MB er-10 k (deep groove ball bearing)
Number of rolling elements	8
Rolling element diameter	7.938 mm
Pitch diameter	33.503 mm
Inner diameter	19.05 mm
Outer diameter	47.96 mm
Motor model	Marathon
Horse Power (hp)	1/3 hp
max. speed	4000 rpm
Shaft diameter	5/8’ turned, ground, polished (TGP) steel
Accelerometer	Kistler 8763a50- Triaxial Sensor
Sensitivity	100 mv/g
DAQ system	Atalon: ata-daq042451
No. of channels	4
Software used	Dewesoft 7.1

## Methodology

### Vibration data collection and preprocessing

In this study, vibration data for DGBBs was collected under various operational conditions using a bearing fault simulator. Speeds of 1000 rpm, 1500 rpm, 2000 rpm, and 2500 rpm were employed to capture data across different fault scenarios, including healthy bearings, inner race faults, outer race faults, ball faults, and combined faults. A tri-axial accelerometer mounted vertically, affixed with bee wax to the bearing housing, recorded the vibrations in three orthogonal directions at the sampling rate of 12,800 Hz. The data was collected using Dewesoft software which was connected to Data Acquisition (DAQ) system and the raw data processed through MATLAB for the subsequent analysis. After that the data was normalised to perform the feature extraction. This step involved scaling the data to a standard range, usually between 0 and 1. Normalization ensured that all features contributed equally to the bearing fault analysis.

### Choice of feature extraction parameters and their relevance to fault diagnosis

The choice of feature extraction parameters for bearing fault diagnosis is based on their ability to capture essential characteristics of the vibration signal. The Mean provides the central tendency, while RMS measures the signal’s power and amplitude, crucial for understanding overall vibration energy. Skewness and Kurtosis measure the asymmetry and peakedness of the signal distribution, indicating non-symmetric faults and the presence of extreme values. Crest Factor, Peak-to-Peak, and Impulse Factor identify high peaks and transient events, which are often associated with defects. Standard Deviation and MAD measure dispersion and variability, providing robust indicators of subtle changes in the signal. Energy quantifies the total power contained in the vibration signal, and Entropy assesses the signal’s complexity, indicating randomness. SNR measures the ratio of signal power to noise power, enhancing the ability to distinguish between faults and noise. Shape Factor provides information about the waveform shape, helping differentiate between various types of faults.

These feature extraction parameters are highly relevant to fault diagnosis as they provide critical insights into the condition of the bearings. Mean and RMS help identify shifts and increased vibration energy, suggesting potential faults. Changes in Skewness and Kurtosis can reveal non-symmetric faults and sudden impacts. Elevated Crest Factor and Peak-to-Peak values indicate periodic impacts and large fluctuations, often due to defects. High Standard Deviation and MAD values signify increased vibration levels and variability, which can be linked to faults. Increased Energy levels can indicate the presence of anomalies. High Entropy values suggest complex, irregular signals associated with abnormal conditions. A lower SNR highlights the presence of noise, potentially masking fault signatures. High Impulse Factor indicates sharp, transient events caused by defects. Shape Factor helps in distinguishing different fault types based on waveform characteristics. Together, these parameters enable a comprehensive analysis for accurate and reliable bearing fault classification.

### Feature parameters

Mean: It is the average value of the dataset. Syntex: np.mean (dataset).

RMS- (Root Mean Square): It is average of squares of dataset, Syntex: np.rms (dataset).

RMS: It provides the Average Line of the Provided data set.

Skewness: It provides an outline for the imported data. If the data is right skewed.

Kurtosis: It checks the max value of the data set; then indicates whether it is impulsive in nature or not.

Crest factor: It takes the second maximum value of the data set through differentiating and comparing and understanding its max and peak value.

Peak2peak: represent range of the data.

Std-Dev: It helped us understand the nature of the data on how the data and the vibration are performing.

Energy: It shows the total energy consumed during the process.

Entropy: It shows the Randomness of the data set.

MAD (Mean Absolute Deviation): It is showing the average of all the presented values with the average.

SNR (Signal-to-Noise Ratio): It measures the strength of the desired signal by noise.

Impulse factor: Its important fault features that detect faults and their characteristics.

Shape factor: Equal to one gives the symmetry of the data.

### Formulas

Mentioned formulas are also utilised as follows.

1 $$MEAN=\Sigma \left(i=1 to R\right){\left(\frac{{\left(yi-mean\right)}^{2}}{R}\right)}^{0.5}$$

2 $$RMS={\left(\frac{{\sum }_{i}^{R}{y}_{i}^{2}}{R}\right)}^{0.5}$$

3 $$crest Factor=\frac{\left|yi\right| \left(Peak value\right)}{RMS}$$

4 $$Form Factor=\frac{RMS}{MEAN}$$

5 $$SHAPE FACTOR=\frac{RMS}{1/R(\Sigma (I=1 TO R)|Yi|}$$

6 $$skewness= \frac{{{\Sigma }_{i=1}^{R}\left({y}_{i}-\underline{y}\right)}^{4}}{R{\sigma }^{4}}$$

7 $$peak to peak \left(Range\right)= y-ymin$$

8 $$Impulse Factor= \frac{mAx\left|{y}_{i}\right|}{\frac{1}{R}{\Sigma }_{i=1|}^{R}{y}_{i|}}$$

### Feature selection

Before selecting features, a labeled dataset containing all features was prepared based on the obtained feature extraction data. In order to bearing fault classification, this was achieved through a process that involved choosing the best subset of features from a raw set to reduce the feature space’s dimensions. The aim was to simplify data structure, enhance data comprehension, and improve the model’s identification, stability, and robustness. Although several methods can determine feature significance scores, the Maximum Relevance Minimum Redundancy (MRMR) approach was chosen. This technique sorted ten features and reduced the subset’s dimensionality.

9 $$I\left(Y,X\right)={\iint }_{\Omega x}^{\Omega y}p\left({x}_{,}y\right)\text{log}\left(\frac{p\left({x}_{,}y\right)}{p(x)p(y)}\right)dxdy$$

X represents the feature set, Y denotes the response variable, ΩY and ΩX are the sample spaces corresponding to Y and X, p(x, y) is the joint probability density, and p(x) is the marginal density function.

The MRMR feature information function:

10 $${f}^{mRMR}\left({X}_{i}\right)=I\left(Y,{X}_{i}\right)-\frac{1}{\left|s\right|}\sum_{x\in x}^{1}I\left({X}_{s},{X}_{y}\right)$$

To achieve this, relevant features correlated to target activities were retained, while irrelevant ones were discarded by analyzing their relevance to target activities. Next, the chosen features’ redundancy was analyzed to reduce the feature subset’s dimensions further. The MRMR algorithm aimed to identify the minimal number of features that could accurately classify inputs. After feature extraction and selection, the essential characteristics for model training were obtained. Based on this, two deep learning models such as Resnet-50 and Alexnet-50 were constructed and trained using the same data. Each model’s accuracy was evaluated to determine the best fit for subsequent fault classification.

### Resnet50 Architecture and its working principle

Input Layer: Instead of 2D images, our input is 1D vibration data with 14 features. This data represents the vibration signals collected from the bearing.

1D Convolutional Layers: The original ResNet-50 uses 2D convolutions, but for 1D vibration data, we replace these with 1D convolutions. These layers apply filters to the 1D data to extract important features over time.

### Residual blocks with 1D convolutions

Each residual block consists of several layers with 1D convolutions, batch normalization, and ReLU activations. Skip connections in these blocks and allow the network to bypass certain layers, mitigating issues like the vanishing gradient problem. This is particularly useful in deeper networks.

ReLU Activation: Introduces non-linearity, helping the network learn complex patterns in the data.

1D Pooling Layers:

1D Max Pooling: Reduces the dimensionality of the data by down-sampling, retaining the most significant features.

1D Average Pooling: Further reduces dimensionality in deeper layers of the network.

Fully Connected Layers: After the convolutional and pooling layers, the extracted features are passed through fully connected layers. These layers integrate the features learned by the convolutional layers.

Output Layer: Softmax Activation: Converts the final scores into probabilities, indicating the likelihood of each class (e.g., different types of bearing faults).

**Fig. 2:**
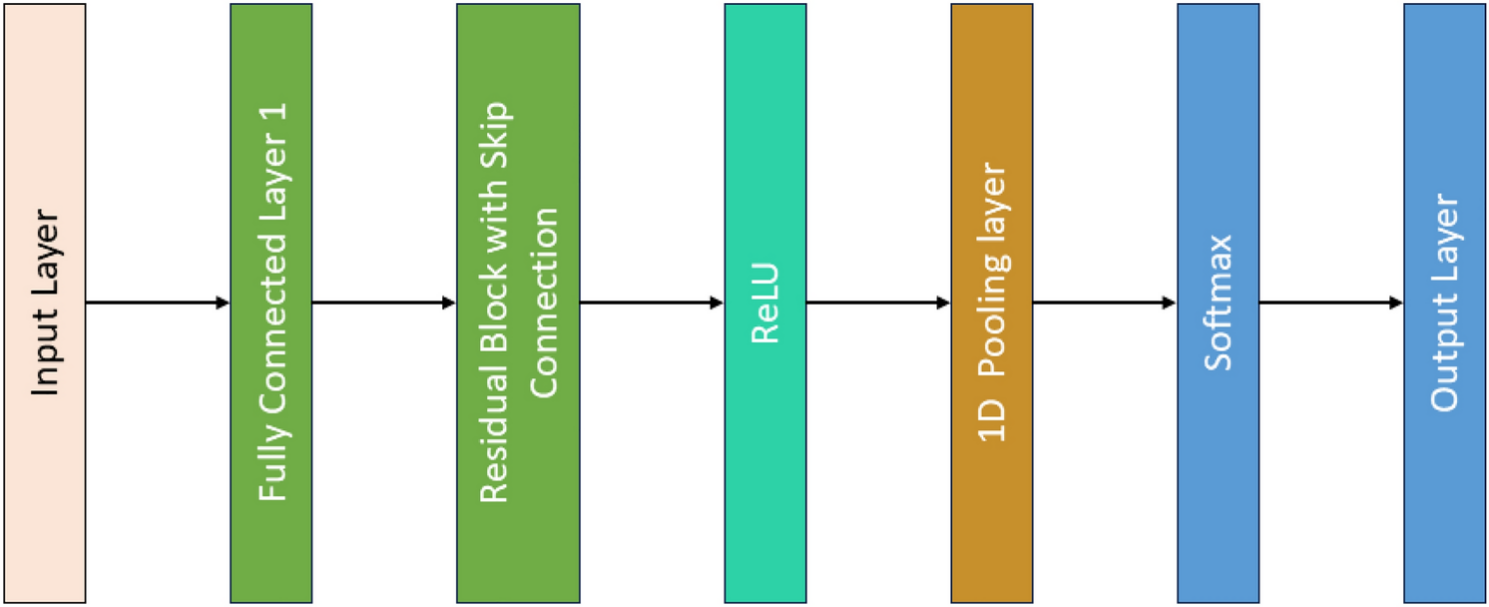
ResNet-50 Architecture

Figure 2 shows the architecture of ResNet50 and it employs a series of 1D convolutional layers followed by residual blocks, which include skip connections. These residual connections facilitate efficient gradient flow and allow the network to learn complex features from the vibration signals. Max-pooling layers are used to reduce the dimensionality of the feature maps while preserving essential information. The learned features are then passed through fully connected layers, and the final classification of bearing conditions (healthy, inner race fault, outer race fault, ball fault, combined fault) is achieved using a softmax output layer.

ResNet50 can be equipped to analyse vibration signal data as time-series or spectrogram images. The residual network’s deep architecture helps in extracting high-level features that are indicative of the condition of ball bearing. The model is trained on labelled data where different conditions (normal, fault, etc.) are the classes.

### Strengths

- Residual blocks help in learning complex patterns in vibration data, which can be non-stationary and noisy.
- Skip connections and allow the model to learn both low and high-level features effectively.
- Residual learning eases the training of deep networks by using shortcut connections, mitigating the vanishing gradient problem, and ensuring efficient gradient flow.
- Captures complex features and patterns, leading to high accuracy in fault diagnosis.
- Supports transfer learning, allowing pre-trained models to be fine-tuned for specific tasks, making it adaptable for various fault diagnosis scenarios.

### Weaknesses

- Requires significant computational resources, which can be a limitation for real-time applications or systems with limited processing power.
- May be prone to overfitting, especially with small datasets.
- Since vibration signals are 1D, the model needs to be adjusted from Conv2D to Conv1D layers, which might affect performance.

### AlexNet50 Architectures and its working principle

Input Layer: This is where the 1 × 1x14 sized input data enters the neural network, transforming the raw data into a format suitable for processing by the following layers.

Fully Connected Layer 1: This layer, consisting of 256 neurons, connects each neuron to every neuron in the input layer, allowing it to learn various patterns and features from the data.

ReLU Activation: ReLU, or Rectified Linear Unit, is an activation function that adds non-linearity to the model. It keeps positive values the same while converting negative values to zero, helping the model learn complex patterns.

Fully Connected Layer 2: This layer includes 128 neurons, each connected to every neuron in the previous layer. It further refines pattern and feature learning.

ReLU Activation: Another ReLU activation function is applied to introduce additional non-linearity, aiding the model in learning more intricate patterns.

Fully Connected Layer: This layer comprises 5 neurons, each representing one of the potential classes for the input classification.

Softmax Activation: Softmax, another activation function, is typically used in the final classification layer. It transforms raw output scores into probabilities that add up to 1, indicating the likelihood of the data belonging to each class.

Classification Output: The final layer provides the classification result. It uses cross-entropy loss to evaluate how well the predicted probabilities align with the actual class labels.

**Fig. 3:**
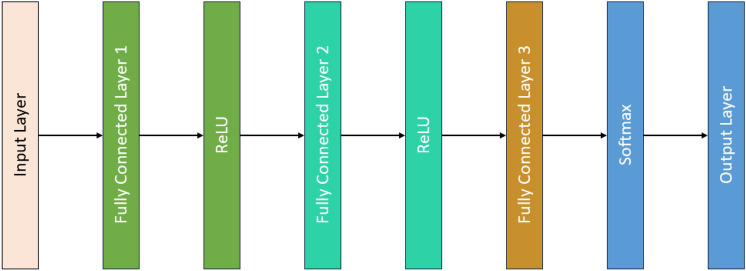
AlexNet-50 Architecture

In Fig. 3 Shows the architecture of AlexNet50, on the other hand, uses multiple 1D convolutional layers with ReLU activation functions to extract features from the vibration data. Following each convolutional layer, max-pooling layers are used to down-sample the features, reducing the computational complexity. Fully connected layer and softmax activation were used for final classification. The hierarchical feature learning approach of AlexNet50 ensures that the network captures both low-level and high-level abstractions from the vibration data. The final fully connected layers consolidate the learned features, and the softmax output layer classifies the different bearing fault conditions.

**Fig. 4:**
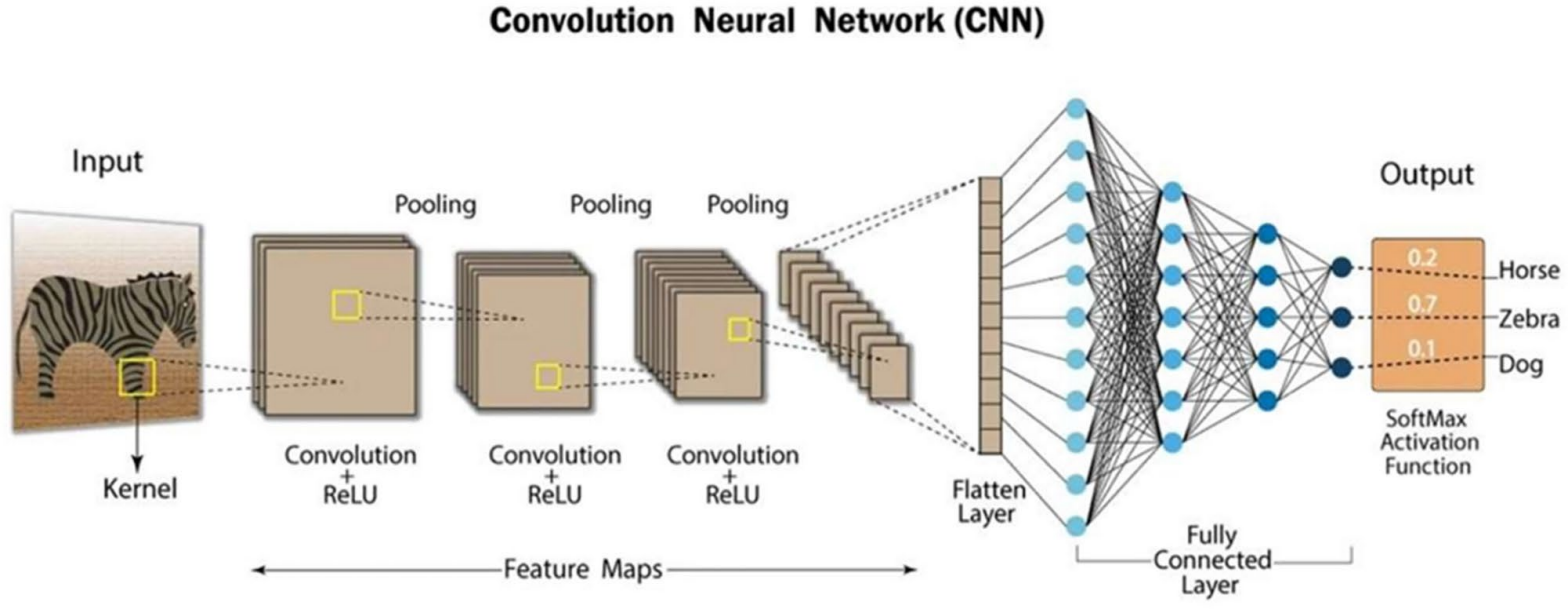
Functioning of a traditional CNN

Alexnet50 is traditionally used for image data which can be adapted to classify vibration patterns by converting vibration signals into spectrograms or any other visual size representations. Now the model layers learn features specific to normal or faulty bearing conditions, helping in accurate classification. Figure 4 shows working of traditional CNN (Convolution Neural Network).

### Strengths

- Simplicity and efficiency with less computationally intensive architecture, making it suitable for applications with limited resources
- Proven performance in various fault diagnosis tasks demonstrates reliable results.
- Convolutional layers are effective in extracting relevant features from vibration signals.

### Weaknesses

- Shallower depth compared to ResNet50 may limit its ability to capture very complex features.
- Larger kernels might not translate well to 1D; using smaller kernels would be better.
- Higher risk of overfitting with fewer layers if the dataset is small.
- Lacks the flexibility of newer architectures in adapting to different fault diagnosis tasks.
- Considered outdated compared to modern architectures that offer better performance and efficiency.

The use of Decision trees can be classified for ball bearing conditions based on extracted features from the vibration data such as amplitude, frequency, statistical metrics (e.g., RMS, Kurtosis). Now the trees split data based on the most informative features that are leading to a prediction of the bearing’s state.

Discriminant analysis (LDA or QDA) can be applied for feature space obtained from vibration signals. It can be assumed that different bearing conditions follow different kinds of probability distributions and attempt to classify the condition given based on the data given.

Logistic regression could be a great way to model the probability of a ball bearing being in a particular state (e.g., faulty Vs normal) based on features extracted from the vibration data. Its output is a probability that can be the threshold to make a classification decision.

Naïve Bayes are analysed through the vibration data by calculating the likelihood of each bearing condition given the features, under the assumption that the feature is independent. It performs well in practice for classification tasks for prediction.

SVM support can be used to classify ball bearing conditions by finding the hyperplane that separates different states of the bearing (Healthy, faulty, etc.) based on the extracted feature from vibration data. Kernels can be applied if the data is not linearly separable.

KNN can be used for ball bearing conditions by comparing its vibration data features with those of the nearest neighbours in the feature space. The condition is determined by the majority class of the nearest neighbour making it a straightforward but powerful method.

Kernels are applied within SVM or other algorithm that has to be project the vibration data into a high level of dimensional space; there it becomes easier to separate classes (Healthy and faulty) Different Kernel functions (RBF, Polynomial) can be experimented with bases of the data characteristics.

Ensemble classification has multiple types to contribute into the system.

Boosted Tree: It combines multiple weak classifiers sequentially on the vibration data to improve accuracy. Each model present in the sequence tries to correct the error of the previous one.

Bagged Tree: It is operating methods such as Random Forest, where multiple decision trees are trained on different subsets of the vibration data. The final decisions are made based on the majority vote for improving robustness.

Subspace Discriminant & KNN Tree: Train multiple models on different random subsets of features extracted from the vibration data, combining their results to enhance accuracy.

RUS Boosted Tree: Handles imbalanced datasets, such as rare bearing fault conditions, which is by under-sampling the majority class and boosting the minority classes representation.

Optimizable Ensemble: Its performance fine-tuning hyperparameters across various ensemble techniques, optimising the model for the best possible classification accuracy on the vibration data.

### Neural network classification

Narrow, Medium, Wide Networks: Depending on the complexity of the vibration data, a neural network having different widths can be utilised. Narrow networks work for simpler patterns, while wider networks are for more complex vibrations.

Bilayered, Trilayered Networks: They allow the model to capture intricate patterns in the vibration data, making it suitable for more complex classification tasks at hand.

Optimizable Neural Network: Now, it involves optimising the given network’s architecture and given hyperparameters that are the best fit for the vibration data. It is Automated Machine Learning (AutoML) techniques.

The MATLAB platform is used for the fault analysis provided for the ball bearing. Extraction of vibration data for one axis out of the three axis is taken. A frame of 10 data points is created for valid calculation of the data. A total of 30 epochs were created that stored about 14,000+ data for both training and testing. This data converts into an array form to be used by classification learner application.

Evaluation for the data through feature retraction was performed and a total of 14 features as input parameters were considered to be taken for the evaluation and inbuilt function was used for performing the formulation.

## Results and discussion

This study contributes to the existing research on fault diagnosis by leveraging advanced deep learning models such as ResNet50 and AlexNet50. Our approach introduces novel techniques in data acquisition, preprocessing and model training, which result in improved diagnostic accuracy. The methodology employed in this research incorporates unique strategies that enhance the performance of these networks, distinguishing our work from the previous studies.

The key contributions of our study are as follows:

Innovative Methodology: Applied convolutional neural networks (CNNs) to vibration signals instead of image-based problems. The transition from 3 to 1D processing results in improved computational time and efficiency. Both ResNet-50 and AlexNet-50 are well-suited to handling large datasets, making it easier to train these deeper models and achieve higher accuracy.

Enhanced Accuracy: Our experiments demonstrate a significant increase in diagnostic accuracy compared with the traditional methods and existing studies using ResNet50 and AlexNet50. This improvement is achieved without struggling with feature representation in higher order data and the model’s fault diagnosis capabilities for 1D vibration signals are improved.

Practical Applications: The findings of this research have practical implications for industries relying on fault diagnosis systems, potentially leading to more reliable and efficient operations.

### Feature ranking measure by MRMR algorithm

**Fig. 5:**
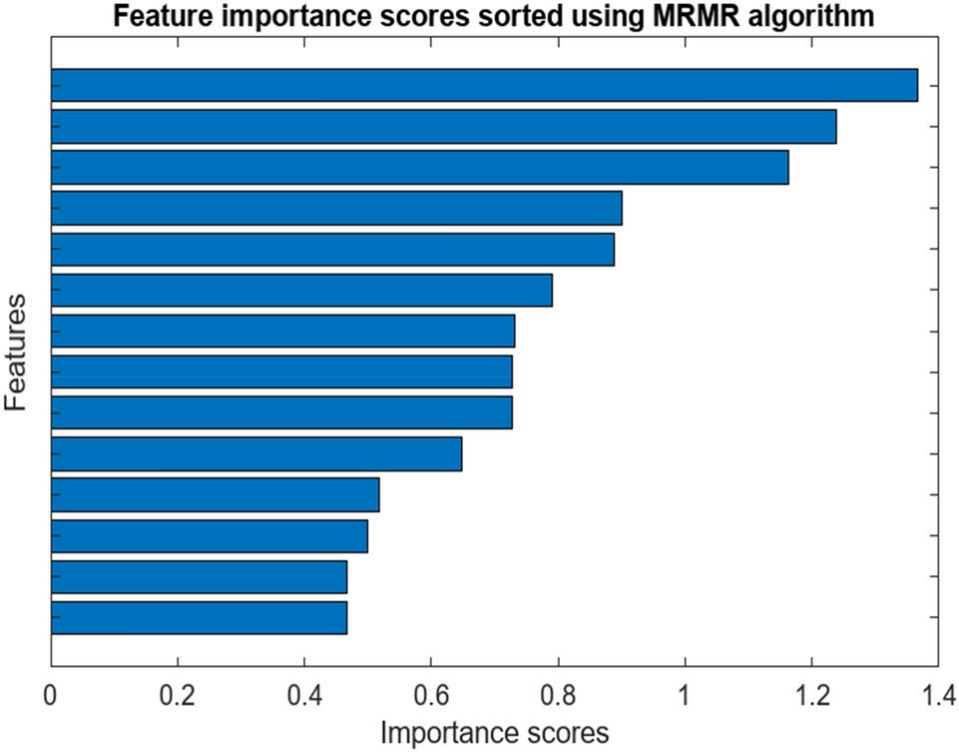
Feature rankings using the MRMR approach

MRMR stands for Minimum Redundancy Maximum Relevance. It helps in balancing the predictive models more effectively. Figure 5 shows selected Ranked features providing information about the contribution for each 14 parameters with values mentioned below.

The highest ranked feature shows the Highest level of information sharing with relevant data.

It Ranked as: 1. Mean- 1.3656, 2. CrestFactor-1.2378, 3.SNR-1.1635, 4. Peak2Peak − 0.9011, 5. Shape factor- 0.8888, 6. MAD—0.7902, 7. Impulse factor- 0.7306. This affected ratio contributed to the formation of the confusion matrix mentioned in the below Fig. Provided 5 labelled classes for faulty data were considered as BF (Ball Fault bearing), CF (Combined Fault bearing), HB (Healthy Bearing), IRF (Inner Race Fault bearing), and ORF (Outer Race Fault bearing).

Enquired data for the classification learner in the MATLAB App section runs the test for all the respective models such as: Decision tree, Discriminant analysis, Logistic Regression Classification, Naïve Bayes classification, Support vector Machine, KNN-(K-Nearest Neighbours), Kernels- Random Forest, Neural Network Classification, Ensemble Classification.

Supporting the above-mentioned models our work utilised algorithms such as Deep Neural Network, and Convolution Neural Network (ResNet50, and AlexNet50). The Minimum Redundancy Maximum Relevance (MRMR) feature selection method was used on ResNet-50 and AlexNet-50, resulting in high accuracy and improved classification results. This method reduced redundancy, enhancing model performance and accuracy. Both models had a short training time of 1 min and 39 s, demonstrating their efficiency in managing large datasets. ResNet-50, with its complex architecture, had a slightly longer training time, showcasing its optimization capabilities. Both models are suitable for practical applications requiring quick training and accurate classification.

### ResNet50

**Fig. 6:**
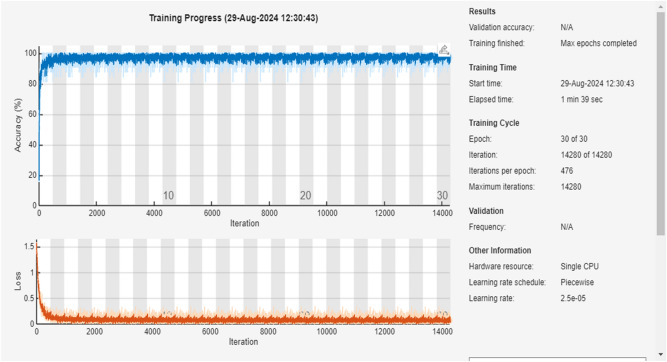
Training process of the ResNet50 model

Resnet50 is considered as a CNN algorithm that works with vibration analysis, providing the highest level of accuracy for a large number of data sets. Figure 6 shows training of the Algorithm that has 14 different feature extraction processes for a course of 5 classes of training data, adding more connected layers for improving the accuracy of the given trained data.

Activation Layer (ReLu) transfers the value of the input neuron into the output Neuron to be passed to the next Layer. ReLu Layers help by maintaining non-Linearity in the dataset.

Dataset Divided into two categories:

1. Training set data: It contains about 80% of the input data.
2. Testing set data: It works on the 20% of the remaining data.

Fully connected Layers add to the Hidden Layer and have Pooling layer for dimensional reduction. This Algorithm is used for providing high value of accuracy due to its deep training with the dataset.

**Fig. 7:**
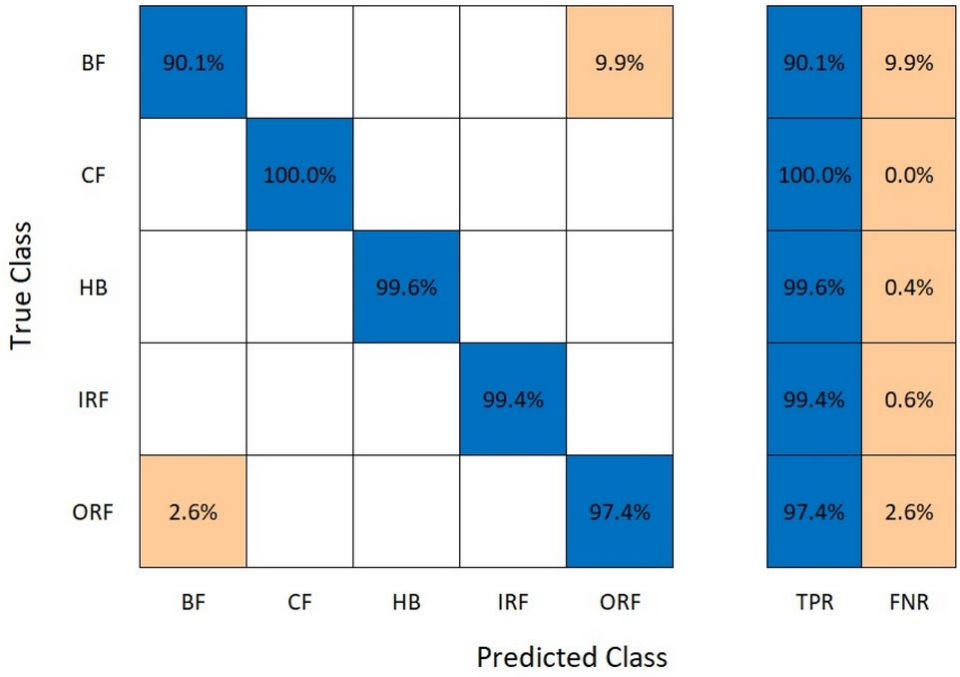
Confusion matrix for ResNet50

Figure 7 illustrates a confusion matrix showing the data that have the common predicted and actual value for test and training. Figures 6 and 7 are the results, shown by 97.9% of the predicted value matching the actual value and about 2.1% of the predicted value; deviate values during the training as its predicted value was different from the result. The ResNet50 algorithm provides connection between neurons at a far deeper level than the alexnet50 algorithm.

Table 2 shows a column for alignment and misalignment that represent accuracy and loss percentage for different labelled classes. The Aligned% shows the correctly matching pairs and vice versa. HB has a high alignment rate with no mismatch. Together including all the classes, the average accuracy is 97.9% and average loss is 2.1% this indicates high accuracy and low loss across all the labelled classes.

**Table 2:** Comparison of alignments for ResNet50.

Class	Aligned (%)	Misaligned (%)
HB	100	–
BF	90.07	9.93
CF	99.59	0.41
IRF	99.37	0.63
ORF	97.37	2.62

### AlexNet50

**Fig. 8:**
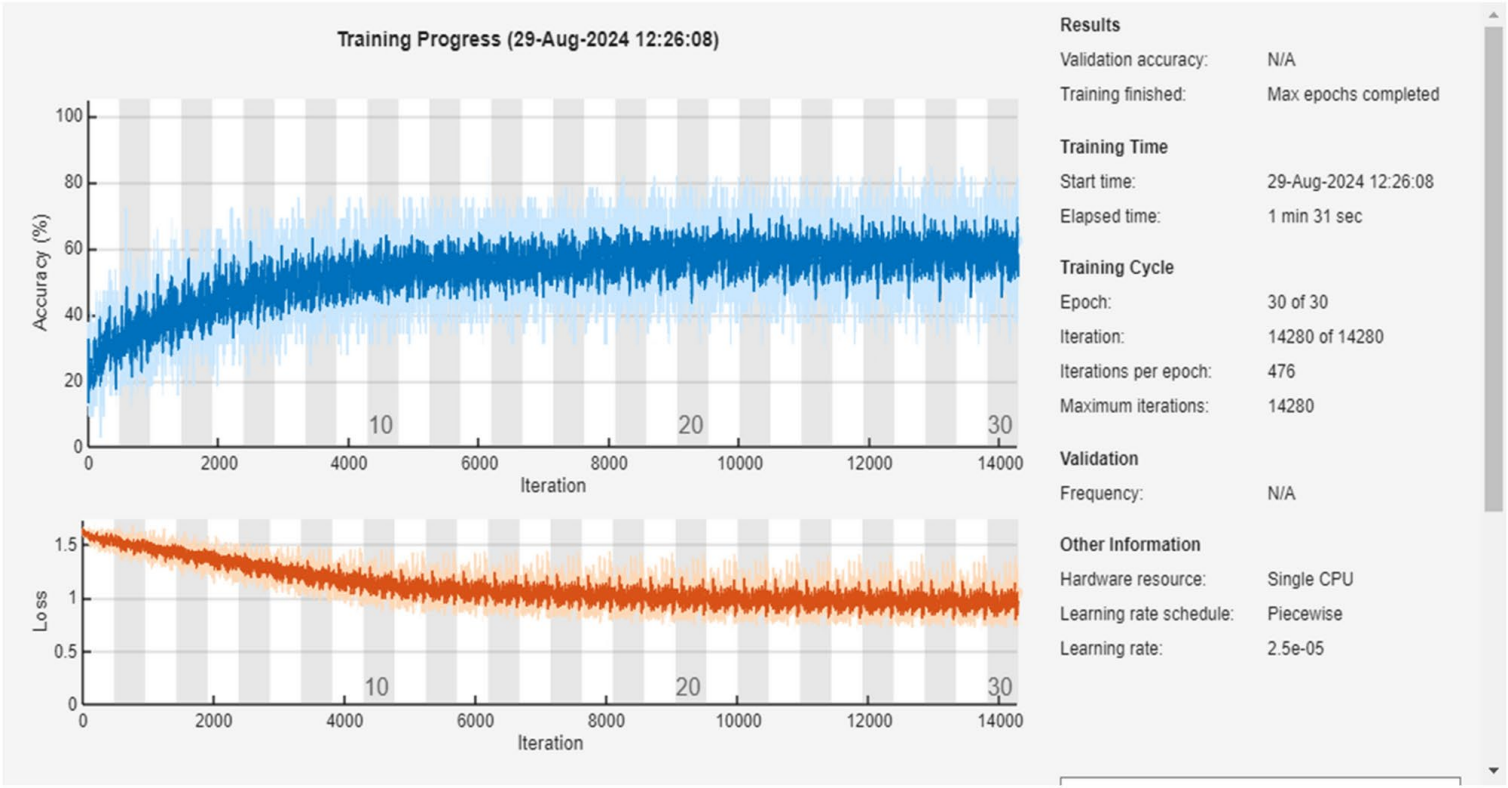
Training process of the AlexNet50 model

Figure 8 is a graph for the training of the algorithm that is operating with 14 different parameters of the feature extraction process, The parameters are having labelled data for training for about five (5) Number of classes.

The input sample counts as 15,246 that is divided into two divisions:

1. Training data: 80% value.
2. Testing data: 20% value.

The computational timing for training the data of provided value is considered low. Additional, hidden layers are introduced concluding 2 Fully Connected Layers and 2 Relu layers for accuracy. This Algorithm when compared with the ResNet50 Algorithm is 0 Seconds faster. The provided algorithm has been used for having Low computational Timing with a large set of data. The error percent ranges from 37.5 to 17% for the testing data.

**Fig. 9:**
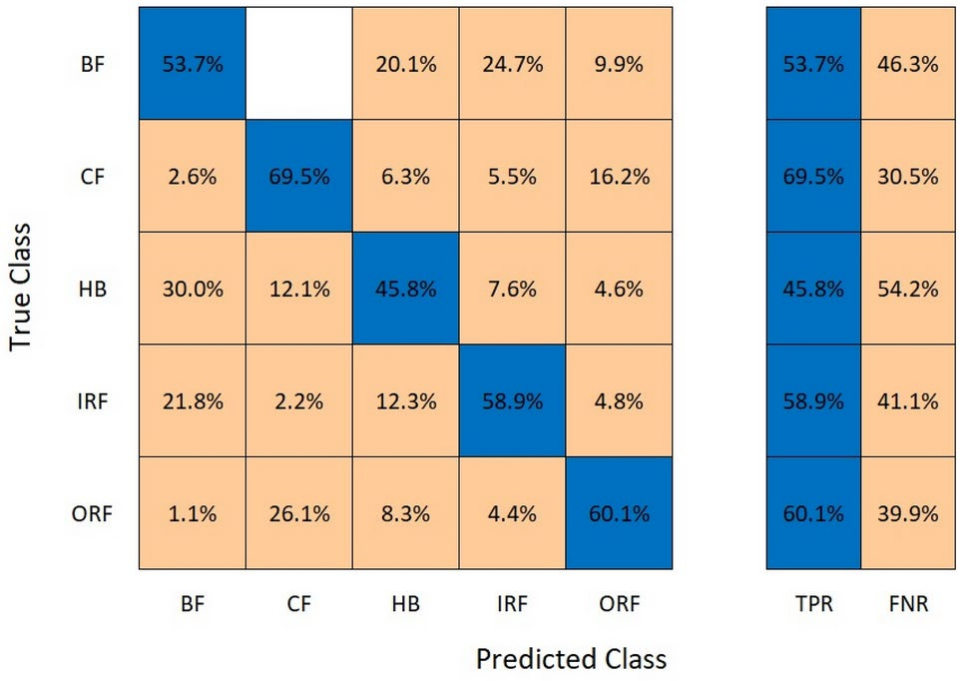
Confusion matrix for AlexNet50.

Provided Figs. 8 and 9 show the result accuracy, above 55% of the predicted values are equal or matching the actual values. The given confusion matrix has the predicted data comparison. Figures 7 and 9 show the confusion matrix for the respective algorithms AlexNet50 and Resnet50, highlighting their accuracy percentages. Figures 6 and 8 show the training graph for these algorithms, the epochs, and computational timing for the respective algorithm and their range of Accuracy and Loss verses iteration are provided for the comparison.

AlexNet50 operated faster by connecting surface neurons without going through the fine filtration process.

Table 3 is the representation of the alignment/misalignment column that provides precision and loss percentage for various classes as labelled. The Aligned% represents the accurate matching pairs and vice versa. HB exhibits a very high alignment. In combination with all the classes, the average accuracy is at 57.58%, while the average loss is 42.81%, showing required accuracy with minimal loss among the labelled classes.

**Table 3:** Comparison of alignments for AlexNet50.

Class	Aligned (%)	Misaligned (%)
HB	69.46	30.54
BF	53.74	46.26
CF	45.77	54.23
IRF	58.86	41.12
ORF	60.08	39.92

**Table 4:** Comparison of alignment for decision tree.

Class	Aligned (%)	Misaligned (%)
HB	100	–
BF	94.4	5.6
CF	100	–
IRF	96.3	3.7
ORF	100	–

### The classifier learner in MATLAB

Working for additional five (5) Algorithms for comparing the accuracy between them. Operation for supervised learning of the process with labelled data was considered. Fourteen (14) parameters from feature extraction methods were used for the comparison between respective models.

The model suggested will work on a noncontact type of maintenance. This is something preferable for the relevant machining industry, as with this model, it becomes easy to study and analyse multiple tools and their parts. The accuracy of the data to be achieved in the long run will dictate the breakdown throughout the installation period.

Vibration data was obtained from the experiment. The design of the self-aligning bearing had been used to execute the empirical mode decomposition Huang transform to give the Huang spectrum of the deep groove bearings.

#### Decision tree

**Fig. 10:**
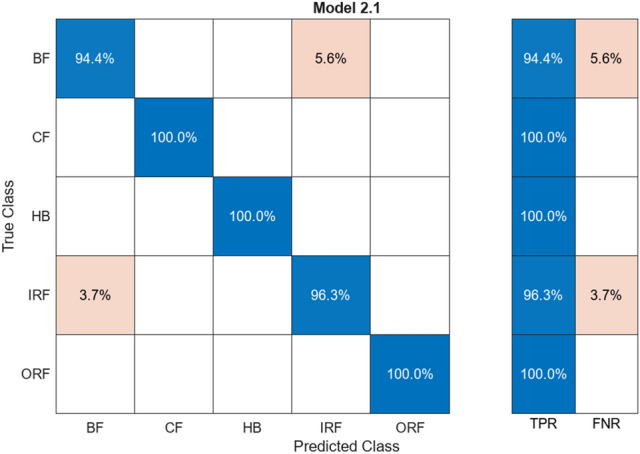
Confusion matrix for Decision Tree

The above figure, Fig. 10, is a confusion matrix that presents the percentage of the matching between training values and testing values. The following matrix is in the range 94–100%. The matrix is assigned to have 5 different classes, as stated in the Y-axis of Fig. 10. The algorithm splits the data with respect to feature extraction. It provides branches that will compare the values. Confusion matrix compares the actual data with the predicted data provided by the ML.

Table 4 The alignment/misalignment column is represented by giving the precision and loss percentage of various classes as labelled. The Aligned% is the number of accurate matching pairs, and vice versa.

HB, CF, ORF have a very high alignment with no mismatch of the data. The average accuracy is at 97.7% with all the classes, with the average loss being 2.3%, which shows an accuracy well-represented with minimal loss among the labelled classes. The extracted features in terms of the amplitude, frequency, and statistical metrics-RMS, Kurtosis-of the vibration data can be classified via Decision Trees.

In this case, the decision trees split up the data according to the most informative features leading up to the prediction of the bearing condition.

#### Discriminant analysis

**Fig. 11:**
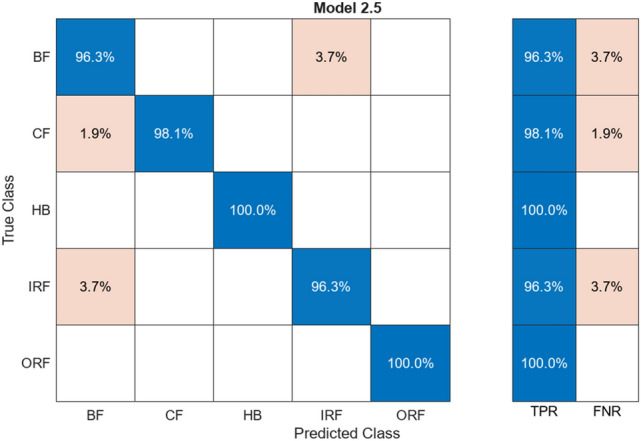
Confusion matrix for discriminant analysis

This algorithm was used as it grouped the imported samples and predicted the categories of dependent variance. The Confusion Matrix finds correlation between the trained and tested data shared by the ML through the fourteen (14) parameters labelled with five (5) classes. Figure 11 provides insight percentage comparison for all the 5 labelled classes. Data comparison between actual value and predicted value is shown in the positive blue marks and misaligned values are marked in the orange.

Table 5 represents a column for alignment and misalignment that displays accuracy and loss percentage for respective labelled classes. Aligned% represents the percentage of matched pairs and vice versa. HB and ORF feature an extremely high alignment rate with no mismatch. Combined in all classes, the overall average accuracy stands at 97.6% while the overall average loss stands at 2.4% implying a very high accuracy as well as low loss throughout the entire labelled classes.

**Table 5:** Comparison of alignment for discriminant analysis.

Class	Aligned (%)	Misaligned (%)
HB	100	–
BF	96.3	3.7
CF	98.1	1.9
IRF	96.3	3.7
ORF	100	–

#### Logistic regression classification

**Fig. 12:**
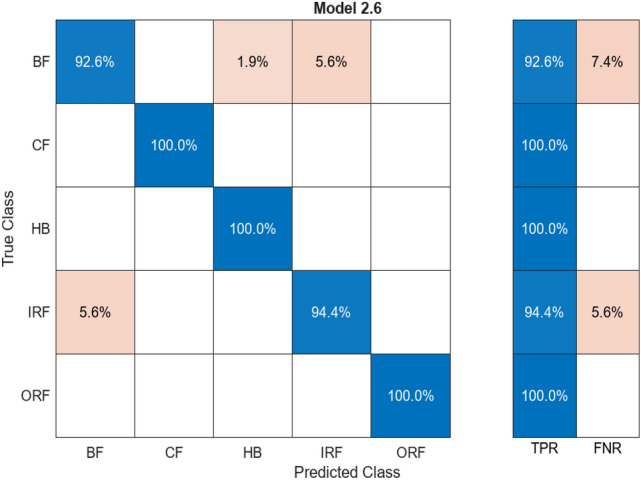
Confusion matrix for logistic regression classification

This Algorithm provides a dependent variable that supports estimating probability and provides a high grade of confidence level for matching prediction. Figure 12 represents Confusion Matrix that is utilised for comparing trained data and testing data provided by the ML. Figure 10 shows percentage comparison for five (5) mentioned labelled classes on the Y axis.

Table 6 illustrates a column for alignment and misalignment that reflects accuracy and loss percentage for corresponding labelled classes. Aligned% denotes matched pairs percentage, which is the percentage of aligned classes. HB and CF show an extremely high alignment rate with no mismatches. Overall average combined in all classes, the average accuracy stands at 97.4% while the average overall loss stands at 2.6%, which gives a very high accuracy as well as low loss throughout the whole labelled classes.

**Table 6:** Comparison of alignment for logistic regression classification.

Class	Aligned (%)	Misaligned (%)
HB	100	–
BF	92.6	7.4
CF	100	–
IRF	94.4	5.6
ORF	100	–

#### Naive Bayes classification

**Fig. 13:**
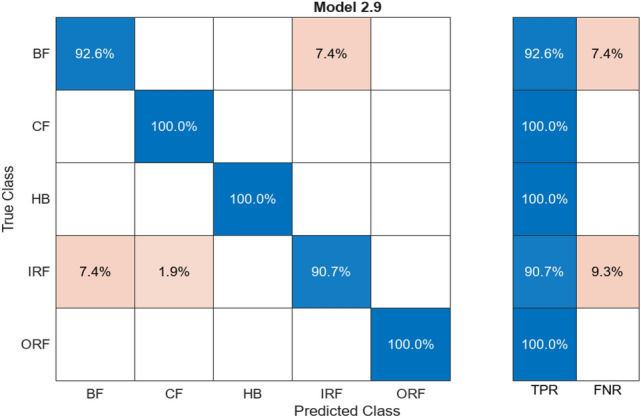
Confusion matrix for Naive Bayes classification

It uses the probabilistic model for the classification of data. Applying Naïve Bayes classification with independent classes also makes its evaluation process simple, and it becomes efficient and fast. The confusion matrix that compares the tested and the resultant data shared by the ML through Fig. 5 MRMR feature extraction. Figure 13 shows percentage comparison for labelled classes.

Table 7 has a column for alignment and misalignment reflecting exactly that particular labelled classes also mirror loss percentage; aligned% depicts the percentage of matched pairs, which is synonymous with how much of all classes is aligned. HB, CF, ORF reflect a very high alignment rate with no mismatch at all. The overall average combined in all classes takes an average accuracy at 96.66% while the average overall loss stands at 3.34%, giving a very high accuracy with low loss throughout the whole labelled classes.

**Table 7:** Comparison alignment for Naive Bayes classification.

Class	Aligned (%)	Misaligned (%)
HB	100	–
BF	92.6	7.4
CF	100	–
IRF	90.7	9.3
ORF	100	–

Naive Bayes is analyzed using vibration data, which computes how probable each condition of the bearings is, given the features under the assumption that the feature is independent. Thus, Naive Bayes works well in practice for classification tasks in prediction.

#### Support vector machine

**Fig. 14:**
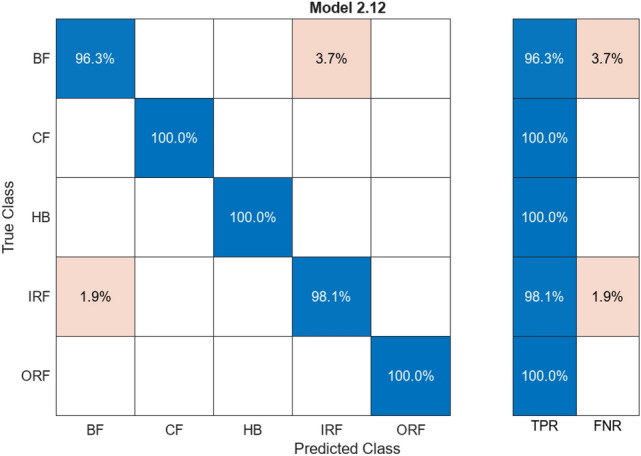
Confusion matrix for Support Vector Machine

A hyperplane separated the classes. A support vector machine finds optimal planes for data separation by the closest data point of each mentioned class by maximizing their marked margins. The confusion matrix was differentiating trained and result data. It was provided through parameters. Figure 14 represents the percentage comparison between the five (5) classes.

Table 8 has an alignment column as well as misalignment reflecting exactly that particular labelled class also reflects loss percentage; aligned% shows the percent of matched pairs, which is equivalent to how much of all classes are aligned. HB, CF, ORF reflect a very high alignment rate without any mismatch at all. The overall average combined in all classes takes an average accuracy of 96.66% while the average overall loss stands at 3.34%, giving very high accuracy with low loss throughout the whole labelled classes.

**Table 8:** Comparison alignment for support vector machine.

Class	Aligned (%)	Misaligned (%)
HB	100	–
BF	96.3	3.7
CF	100	–
IRF	98.1	1.9
ORF	100	–

#### KNN (k nearest neighbor)

**Fig. 15:**
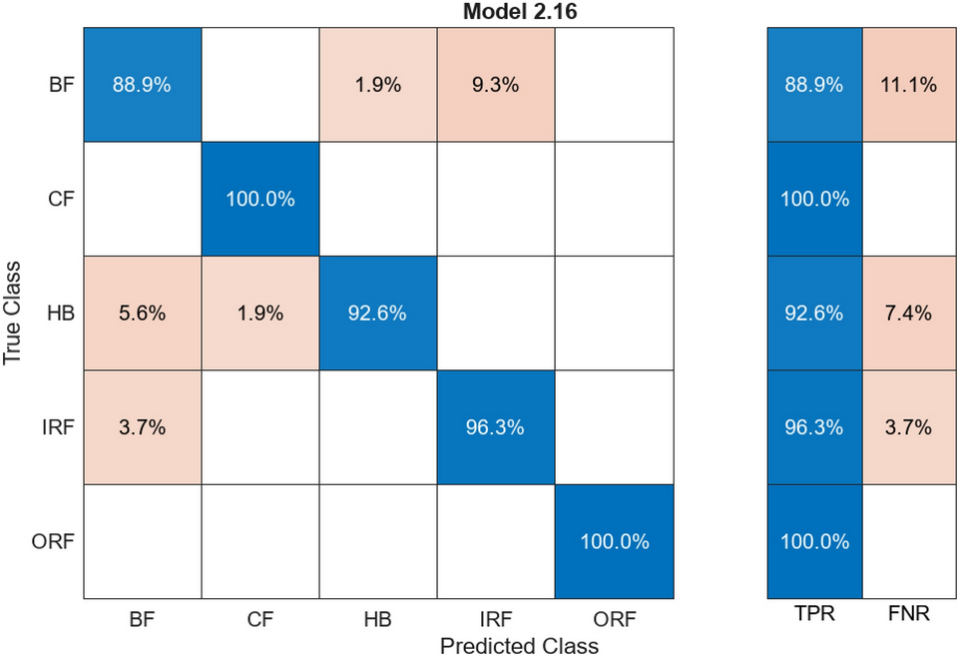
Confusion matrix for KNN

The nearest K neighbors for data points and get assigned to the majority class of neighbors. We used the distance metric and took the distances for each data point by Euclidean distance and Manhattan or Hamming distance. The confusion matrix checks the trained data and the tested data by machine learning. Figure 15 has a percentage comparison with all the 5 mentioned labelled classes.

Table 9 has an alignment column as well as misalignment reflecting exactly that particular labelled class also reflects loss percentage; aligned% shows the percent of matched pairs, which is equivalent to how much of all classes is aligned. CF and ORF reflect a very high alignment rate without any mismatch at all. All class averages combine the average accuracy of about 95.56%, while the average overall loss stands at 4.44%, giving very high accuracy with low loss throughout the whole labelled classes.

**Table 9:** Comparison of alignment for KNN.

Class	Aligned (%)	Misaligned (%)
HB	92.6	7.4
BF	88.9	11.1
CF	100	–
IRF	96.3	3.7
ORF	100	–

#### Kernels-random

**Fig. 16:**
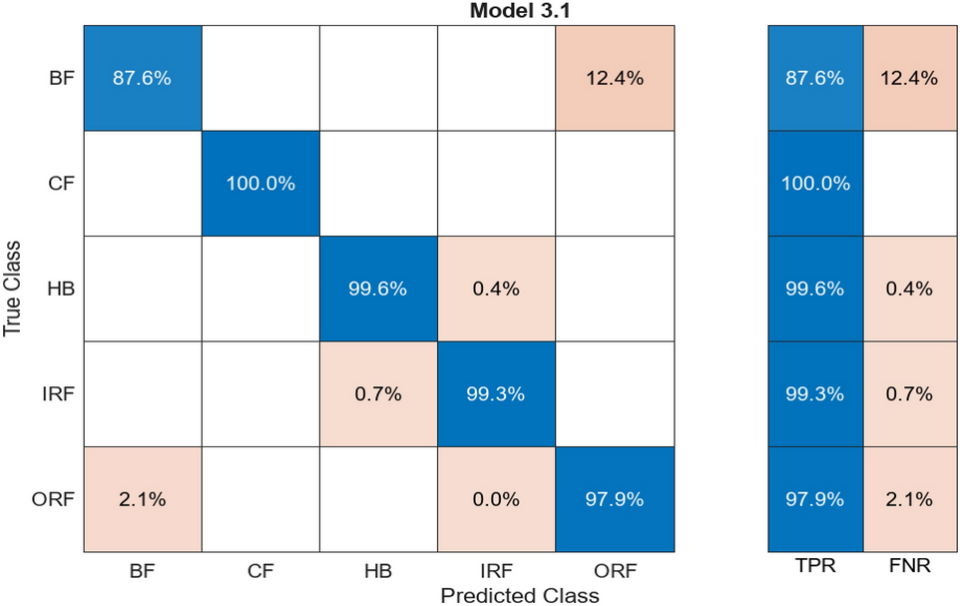
Confusion matrix for Kernels-Random

Kernels random likely has a distinct way of approaching. Connection between random forest and kernel method, where the operation of vectors takes place, handles complex and non-linear matrices and relationships between data. The confusion matrix contrasts between trained data and resulting data shared by the ML resulting from the training. Figure 16 represents percentage differences for the five mentioned labelled classes.

Table 10 has alignment columns and misalignment reflecting exactly that particular labelled class also reflects loss percentage; aligned% shows the percent of matched pairs, which is equivalent to how much of all classes is aligned.

**Table 10:** Comparison of alignment for Kernels-Random.

Class	Aligned (%)	Misaligned (%)
HB	99.6	0.4
BF	87.6	12.4
CF	100	–
IRF	99.3	0.7
ORF	97.9	2.1

CF reflects a very high alignment rate without any mismatch at all. All class averages combine the average accuracy of about 96.88%, while the average overall loss stands at 3.12%, giving very high accuracy with low loss throughout the whole labelled classes.

Kernels are used within SVM or any other algorithm that requires the vibration data to be mapped in a high-dimensional space where it can easily be separated based on classes, in this case, healthy and faulty. Various kernel functions, such as RBF and polynomials, can be experimented with regarding the characteristics of the data.

#### Ensemble classification

**Fig. 17:**
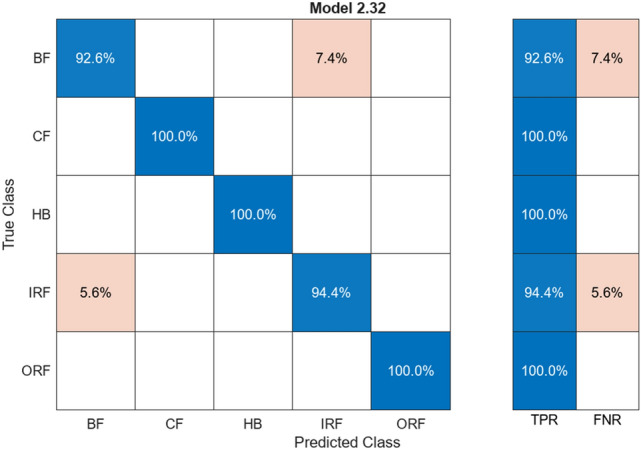
Confusion matrix for Ensemble Classification

The combination provided for their predictions was utilized in the classification of unseen instances operating some form of voting. It helped by eliminating the duplication of the data. The confusion matrix was comparing the imported data and the output data given by the ML gained through the feature extraction process performed with five labelled classes and fourteen (14) varying parameters. Figure 17 shows a percentage comparison for given classes.

Table 11 is accompanied by alignment columns and misalignment reflecting precisely that very same labeled classes also reflect loss percentage; Aligned% shows the percent of matched pairs, which is equivalent to how much of all classes is aligned. HB, CF, and ORF reflect a very high alignment rate without any mismatch at all. All the class averages contain about an average accuracy of 97.4%, and the average overall loss is placed at 2.6%, thus providing very high accuracy with low loss all over the whole labelled classes (Table 12).

**Table 11:** Comparison of alignment for ensemble classification.

Class	Aligned (%)	Misaligned (%)
HB	100	–
BF	92.6	7.4
CF	100	–
IRF	94.4	5.6
ORF	100	–

**Table 12:** Comparison of alignment for Neural Network Classification.

Class	Aligned (%)	Misaligned (%)
HB	99.4	0.6
BF	89.4	10.6
CF	100	–
IRF	99.3	0.7
ORF	96.8	3.2

#### Neural network classification

**Fig. 18:**
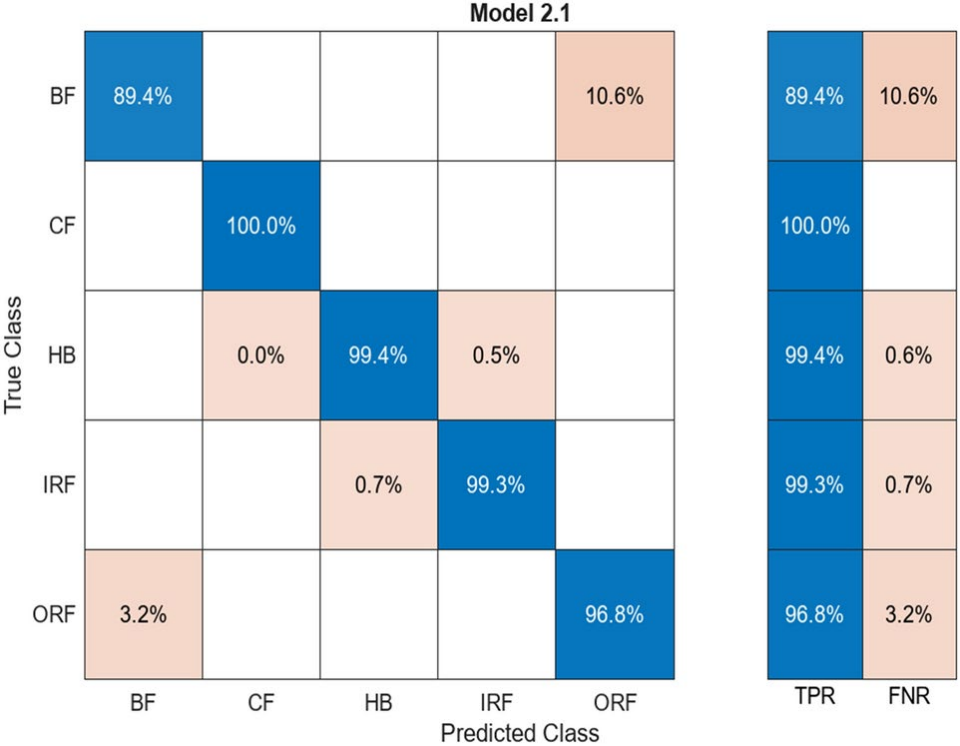
Confusion matrix for neural network classification

Neural Network Classification has a response with the highest output value provided. The confusion matrix was checking the trained data and the test data shared by the ML. Figure 18 shows a percentage comparison with the mentioned five (5) labelled classes.

It is paired with alignment columns and misalignment reflecting just that very same labelled classes also reflect loss percentage; Aligned% shows the percent of matched pairs, which is equivalent to how much of all classes are aligned. HB, CF, ORF reflects a very high alignment rate without any mismatch at all. All the class averages contain about an average accuracy of 96.98%, and the average overall loss is placed at 3.02%, thus providing very high accuracy with low loss all over the whole labelled classes.

### Accuracy comparison between the algorithms

All these models are accompanied by the confusion matrices with accuracy values. The figures as well as the tables accompanying this have units of measurement for the TPR (true positive rate), FNR (false negative rate), accuracy, and loss all in percentage units. It displays the percentage value difference between the training data and test data of all the algorithms used and discussed.

**Fig. 19:**
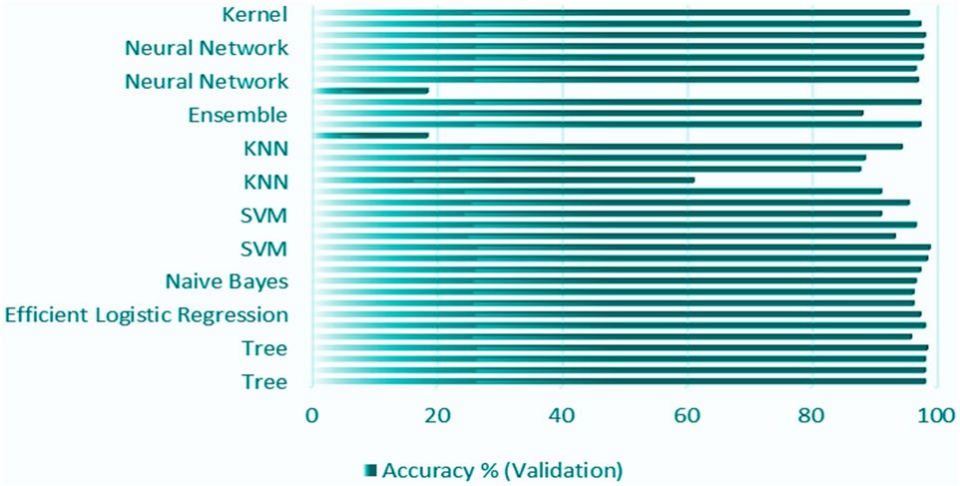
Comparison of accuracy for the algorithm

Figure 19 shows accuracy comparison for the algorithm given by the classification learner in the percentage type format. The ANN algorithm provides accuracy of around 95%+. The CNN algorithm provided in this case has a high-grade accuracy of 97%. Range of Percentage for the classification learner followers is 95–97% percent, but the max output with the confidence level of accuracy is shown in Figs. 4 and 5. It shows an average of 97.87% accuracy for the CNN algorithm with training for 80% of the data and testing the model for 20% of the remaining data set.

The ResNet50 and AlexNet50 algorithms are DNNs (deep neural networks), and the remaining of all the mentioned algorithms, like Random Forest, etc., are ANN representing computational time and accuracy for the deep neural network DNN algorithm, provided random forest (RF), SVM, neural network, and ensemble perform well with complex datasets, as shown by the accuracy. The methods such as decision tree, logistic regression, and discriminant are counted for algorithms that can be interpreted while comparing with the methods like neural networks and support vector machines.

The Depth of Neural Network follows the method as ranked:

1. Low: Logistic Regression, Naive Bayes, Decision Tree, Discriminant Analysis (LDA/QDA), KNN.
2. Medium: Support vector machine, random forest, ensemble classification, neural network.
3. Deep: AlexNet50, ResNet50, Neural Network (depends on the model)

## Main findings

### Section 1: Findings

#### ResNet50

ResNet50 is a CNN algorithm that works in vibration analysis, providing high accuracy across large datasets. Training involved 14 different feature extraction processes over five classes of training data. The addition of more connected layers improved the accuracy. The activation layer (ReLU) maintained non-linearity in the dataset. The dataset was divided into 80% training data and 20% testing data. ResNet50’s architecture led to a high accuracy of 97.9%, with only 2.1% deviation in predicted values during training. It effectively connected neurons at a deeper level than AlexNet.

#### AlexNet50

AlexNet50 showed efficient training performance with low computational timing. Despite its shallower depth, AlexNet50 achieved fast training times and demonstrated adequate accuracy for simpler fault patterns. The dataset was similarly divided into 80% training data and 20% testing data. AlexNet50’s error percent ranged from 37.5 to 17% for testing data, emphasizing its suitability for low computational timing tasks with large datasets.

#### Other algorithms

Algorithms like Decision Tree, Discriminant Analysis, Logistic Regression, Naïve Bayes, SVM, KNN, Random Kernels, Ensemble, and Neural Network Classification all demonstrated high accuracy, generally above 95%. They were effective in extracting relevant features from vibration data and showed high alignment between predicted and actual values.

### Section 2: Comparing Algorithm Performance

#### ResNet50 vs AlexNet50

- Accuracy: ResNet50 achieved a higher accuracy of 97.9% whereas AlexNet50’s error percent varied from 37.5 to 17%.
- Complexity: ResNet50’s deeper architecture allows it to capture complex features but requires more computational resources. AlexNet50, with a shallower architecture, is simpler and faster but may struggle with complex fault patterns.
- Training Time: AlexNet50 is faster than Restnet50 training by 10 s, making it more suitable for real-time applications.
- Transfer Learning: Both algorithms benefit from transfer learning, with pre-trained models that can be fine-tuned for specific tasks.

#### Other algorithms

- Decision Tree and Discriminant Analysis: Both algorithms had high accuracy and provided a clear split based on feature extraction, demonstrating reliability.
- Logistic Regression: Provided high alignment and low loss percentages, assuming a linear relationship between features.
- Naïve Bayes: Demonstrated efficiency and fast evaluation with a high alignment rate, assuming independence between features.
- SVM: High accuracy with some computational intensity for large datasets.
- KNN: Effective but performance can degrade with high-dimensional data.
- Random Kernels: Provided high accuracy but required careful selection of kernel functions for complex relationships.
- Ensemble and Neural Network: High accuracy with effective feature extraction but required careful parameter tuning.

These results demonstrate the efficiency of these models in terms of training time and accuracy rate. Machine learning algorithms are often evaluated by their training durations. KNN and Naive Bayes have the shortest training times, making them ideal for quick training. Logistic Regression and SVM fall in the middle, taking 0.302 and 0.296 min, respectively. Ensemble methods like RUS Boosted and Optimizable Ensemble demand the longest training times due to their complexity. ResNet50 took 1 min and 39 s to train, while AlexNet50 required 1 min and 30 s. If training time is imperative, KNN, Naive Bayes, and Discriminant Analysis are the best options. However, it is important to also consider other factors such as model accuracy. In this study, ResNet50 and AlexNet50 are preferable for bearing fault classification based on their accuracy and training time.

### Section 3: Limitations

#### ResNet50

- Resource Intensive: Requires significant computational resources, limiting its practicality for real-time applications or systems with limited processing power.
- Complexity: The deep architecture necessitates longer training times and careful hyperparameter tuning to avoid overfitting, especially with small datasets.
- 1D Data Adjustment: Needs conversion from Conv2D to Conv1D layers for vibration signals, but this may not significantly impact performance.

#### AlexNet50

- Shallower Architecture: May not capture very deep features as effectively as more complex models like ResNet50.
- Overfitting Risk: Higher risk of overfitting with fewer layers if the dataset is small.
- Outdated Design: Lacks the flexibility and efficiency of newer architectures, potentially limiting performance.
- Kernel Size: Larger kernels might not translate well to 1D data; smaller kernels could be more effective.

#### Other Algorithms

- Decision Tree and Discriminant Analysis: May not handle very complex data as effectively as neural networks.
- Logistic Regression: Limited by the assumption of a linear relationship between features.
- Naïve Bayes: Assumes independence between features, which might not always hold true.
- SVM: Computationally intensive for large datasets.
- KNN: Performance can degrade with high-dimensional data.
- Random Kernels: Requires careful selection of kernel functions for handling complex and non-linear relationships.
- Ensemble and Neural Network: Require significant computational resources and careful parameter tuning to avoid overfitting.

This approach can potentially be generalized to other types of bearings or machinery beyond the specific set-up described in the study. The primary factors that influence the applicability include the speed, load conditions, and the operational environment of the bearings. Additionally, cost considerations and the type of bearings pre-installed in the machinery also play a significant role.

The process involves collecting vibration signals from the bearing’s environment and classifying these signals based on the type of fault they indicate. Since the input is based on the environmental conditions around the bearing, this methodology can be adapted to different types of bearings and machinery. Thus, while the specific set-up in the study is a starting point, the approach has the flexibility to be applied to other systems as well.

## Conclusion

The ResNet50 algorithms showed the highest level of accuracy, which is 97.6%; all of the remaining algorithms were having around 96–97% level of accuracy. The data was subdivided into 5 labelled classes of dataset called BF, CF, HB, ORF, and IRF. This dataset was divided into an equal number of training data. The matrices formed with the respective algorithm were very accurate and precise for the ResNet50 algorithm, as it was shown having a computational time of nearly about 1 min that is far better when compared with the rest of the classifier learner algorithm. If it is for the classification learner, it operates with 9 types of algorithms that work independently for calculating the accuracy with all the learner types and their result all the percentages. The given 9 algorithms were near 97% of aligning accuracy of the testing data with the imported data set that proved the code layers and value collected with the triaxial accelerometer were highly precise. Therefore, the algorithm followed patterns for data tracking. All the methods involved can be used for industries where machining operations are held. This machine for bearing will have a life cycle provided by the consumer for safety but can be utilized at long time. This model establishes regulations for post-lifetime usage, helping to prevent major catastrophes that could halt production lines due to minor parts.

## Scope of future work

AI models like Artificial Neural Networks (ANN) and Deep Neural Networks (DNN) are promising for predictive maintenance in industrial applications. These algorithms are ideal for non-contact-based maintenance, ensuring minimal disruption. As part of the industry 4.0 revolution, AI-based predictive maintenance will evolve with advanced machine learning techniques and real-time data analysis. This will enable proactive maintenance issues, optimize operational efficiency, and reduce costs. The integration of IoT and AI technologies will drive a new era of intelligent maintenance systems, ensuring high performance and reliability in industries.

## Data Availability

The datasets generated and/or analysed during the current study are not publicly available due to institution norms but are available with the corresponding author on reasonable request.

## References

[1] An, B., Ha, Y., Lee, Y., Kwak, W., Lee, Y. (2024). Investigation on feature attribution for remaining useful life prediction model of cryogenic ball bearing. In: Chu, F., Qin, Z. (eds) *Proceedings of the 11th IFToMM International Conference on Rotordynamics. IFToMM 2023*. *Mechanisms and Machine Science*, vol. 139. Springer, Cham. 10.1007/978-3-031-40455-9_25

[2] Cheng, R. C. & Chen, K. S. Ball bearing multiple failure diagnosis operating feature-selected autoencoder model. *Int. J. Adv. Manuf. Technol.* **120**, 4803–4819. 10.1007/s00170-022-09054-x (2022).

[3] Cheng, H., Zhang, Y., Lu, W. & Yang, Z. A bearing fault diagnosis method based on VMD-SVD and fuzzy clustering. *Int. J. Pattern Recognit. Artif. Intell.* **33**, 12 (2019).

[4] Chen, J. et al. Medical image segmentation and reconstruction of prostate tumor based on 3D AlexNet. *Comput. Methods Programs Biomed.* **200**, 105878. 10.1016/j.cmpb.2020.105878 (2021). 33308904 10.1016/j.cmpb.2020.105878

[5] Dogan, B. K. et al. Vibration analysis of a hybrid polymer ball bearing with 3D-printed races. *J. Vib. Eng. Technol.*10.1007/s42417-023-01204-z (2023).

[6] Gulati, K., Tiwari, S., Basandrai, K., Kamat, P. (2022). Predictive maintenance of bearing machinery operating MATLAB. In: Saraswat, M., Sharma, H., Balachandran, K., Kim, J.H., Bansal, J.C. (eds) *Congress on Intelligent Systems.**Lecture Notes on Data Engineering and Communications Technologies*, vol. 111. Springer, Singapore. 10.1007/978-981-16-9113-3_10

[7] Harris, T. A. & McCool, J. I. On the accuracy of rolling bearing fatigue life prediction. *ASME. J. Tribol.* **118**(2), 297–309. 10.1115/1.2831299 (1996).

[8] Kannan, P., N. S. Ball bearing fault by feature extraction and fault diagnosis method based on AI ML Algorithms. In *2022 6th International Conference on Intelligent Computing and Control Systems (ICICCS),* Madurai, India, pp. 1310–1314 (2022). 10.1109/ICICCS53718.2022.9788155

[9] Kolodziejczyk, T., Toscano, R., Fouvry, S. & Morales-Espejel, G. Artificial intelligence as efficient technique for ball bearing fretting wear damage prediction. *Wear* **268**(1–2), 309–315. 10.1016/j.wear.2009.08.016 (2010).

[10] Kundu, P., Chopra, S. & Lad, B. K. Multiple failure behaviors identification and remaining useful life prediction of ball bearings. *J. Intell. Manuf.* **30**, 1795–1807. 10.1007/s10845-017-1357-8 (2019).

[11] Liu, J., Xu, Y. & Pan, G. A combined acoustic and dynamic model of a defective ball bearing. *J. Sound Vib.* **501**, 116029. 10.1016/j.jsv.2021.116029 (2021).

[12] Maria Baldeon Calisto, S. & Lai-Yuen, S. K. AdaEn-Net: An ensemble of adaptive 2D–3D fully convolutional networks for medical image segmentation. *Neural Netw.* **126**, 76–94. 10.1016/j.neunet.2020.03.007 (2020). 32203876 10.1016/j.neunet.2020.03.007

[13] Panthakkan, A. K., Anzar, S. M., Jamal, S. & Mansoor, W. Concatenated Xception-ResNet50—A novel hybrid approach for accurate skin cancer prediction. *Comput. Biol. Med.* **150**, 106170. 10.1016/j.compbiomed.2022.106170 (2022). 37859280 10.1016/j.compbiomed.2022.106170

[14] Reddy Kuruvalli, L., Morabad, D., Vijay, H.M., Ratheesh, P. AI-Enabled fault detection for predictive maintenance of ball bearings. In: Gunjan, V.K., Zurada, J.M. (eds) *Proceedings of 4th International Conference on Recent Trends in Machine Learning, IoT, Smart Cities and Applications. ICMISC 2023. Lecture Notes in Networks and Systems*, Vol. 873. Springer, Singapore (2024). 10.1007/978-981-99-9442-7_49

[15] Seera, M., Wong, M. L. D. & Nandi, A. K. Classification of ball bearing faults operating a hybrid intelligent model. *Appl. Soft Comput.* **57**, 427–435. 10.1016/j.asoc.2017.04.034 (2017).

[16] Sierra, C. & Andrea, E. Predictive maintenance techniques. In: *Mining Maintenance. Topics in Mining, Metallurgy and Materials Engineering.* Springer, Cham (2024). 10.1007/978-3-031-59450-2_9

[17] Wang, M., Yan, K., Zhang, X., Zhu, Y. & Hong, J. A comprehensive study on dynamic performance of ball bearing considering bearing deformations and ball-inner raceway separation. *Mech. Syst. Signal Process.* **185**, 109826. 10.1016/j.ymssp.2022.109826 (2023).

[18] Xie, F. et al. Fault diagnosis of rolling bearings in agricultural machines operating SVD-EDS-GST and ResViT. *Agriculture* **14**, 1286. 10.3390/agriculture14081286 (2024).

[19] Zhang, S., Zhang, S., Wang, B. & Habetweenler, T. G. Deep learning algorithms for bearing fault diagnostics—a comprehensive review. *IEEE Access* **8**, 29857–29881. 10.1109/ACCESS.2020.2972859 (2020).

[20] Umar, M., Siddique, M. F., Ullah, N. & Kim, J. M. Milling machine fault diagnosis using acoustic emission and hybrid deep learning with feature optimization. *Appl. Sci.* **14**(22), 10404. 10.3390/app142210404 (2024).

